# Ten years later: A portrait of the implementation of the advanced access model in Quebec

**DOI:** 10.1177/08404704231181676

**Published:** 2023-06-16

**Authors:** Mylaine Breton, Isabelle Gaboury, Christine Beaulieu, Nadia Deville-Stoetzel, Elisabeth Martin

**Affiliations:** 1 198734 Université de Sherbrooke, Longueuil, Quebec, Canada.

## Abstract

Since 2012, implementation of the advanced access model in primary care has been highly recommended across Canada to improve timely access. We present a portrait of the implementation of the advanced access model 10 years after its large-scale implementation across the province of Quebec. In total, 127 clinics participated in the study, with 999 family physicians and 107 nurse practitioners responding to the survey. Results show that opening schedules for appointments over a period of 2 to 4 weeks has largely been implemented. However, reserving consultation time for urgent or semi-urgent conditions was implemented by less than half and planning supply and demand for 20% or more of the upcoming year by fewer than one fifth of respondents. More strategies need to be put in place to react to imbalances when they occur. We demonstrate that strategies based on individual practice change are more often implemented than those requiring changes at the clinic.

## Introduction

Access to primary care is a high priority for the population, healthcare professionals, and decision-makers alike.^1^ Timely access, such that patients can access the care when they need it, is a cornerstone of efficient primary care and a key pillar of the Patient-Centred Medical Home model.^2^ Timely access has remained a major challenge across Canada for the last two decades and is a persistent problem in primary care in several countries.^3^ A recent international report documenting primary care access found that Canada ranks poorly compared to other high-income countries; only 41% of Canadians reported being able to get a same- or next-day appointment the last time they needed medical attention.^4^

The advanced access model is the most recommended innovation around the world that aims to improve timely access and support patients’ needs for relational and informational continuity.^5,6^ Originally developed in the United States in 2002, the model has existed in the primary care literature for more than two decades. It is endorsed by the US Institute for Healthcare Improvement as well as the Royal College of General Practitioners in the United Kingdom.^7^ The effectiveness of the model has been demonstrated in various healthcare systems.^8^ Benefits of advanced access include reduced wait times and missed appointments and improved professional and patient satisfaction as well as provider productivity.^9^

Since 2012, the advanced access model has been widely promoted by the College of Family Physicians of Canada as well as several other provincial organizations and medical associations. Several guides and training opportunities have been developed across the country, namely, by the College of Family Physicians of Canada, the British Columbia and Manitoba ministries of health, and Health Quality Ontario. In Quebec, advanced access has been widely promoted by the Association of Family Physicians of Quebec (*Fédération des médecins omnipraticiens du Québec*; FMOQ), as well as by the Ministry of Health and Social Services (MHSS). Since 2014, an implementation guide has been produced and disseminated on-line by the FMOQ. On-line training modules (six hours of content) have also been made available. Additionally, three-day training cohorts over several months were offered to family physicians between 2014 and 2017. Those first cohorts were offered in various regions and facilitated by family physician advanced access experts from the FMOQ and managers from the MHSS. Physicians were paid and invited to attend with an administrative agent or a nurse on day one. In subsequent years, cohorts were also opened to nurse practitioners.

Soon after national efforts began to engage physicians in this new model of organizing care, nurse practitioners followed in implementing the model, as their practices resemble those of physicians in many regards. In order to standardize advanced access practices and optimize interprofessional collaboration in primary care to ensure timely access to all team members, other types of professionals, such as registered nurses, social workers and pharmacists, have recently started to adapt and implement the model.^10,11^

Despite recurrent problems of access, the advanced access model is still being promoted to improve timely access in primary care in several provinces, such as Quebec, Manitoba, and New Brunswick, with varying anecdotal levels of implementation. The main objective of this article is to present a portrait of the implementation of strategies rooted in the principles of the advanced access model by family physicians and nurse practitioners 10 years after its wide-spread introduction across the province of Quebec.

## Methods

We conducted a cross-sectional study based on an open e-survey hosted on a web platform specifically designed for the purpose of measuring the implementation of advanced access.^1^ As part of the recruitment process for a quality improvement trial (described elsewhere),^12^ the first two authors of this article made several presentations to regional and provincial boards. This led to the opportunity to distribute the e-survey among 127 Family Medicine Groups (FMGs) across Quebec that showed interest in the project. FMGs are groups of physicians working closely with other primary care professionals to provide services to enrolled patients on a non-geographic basis.^13^ Since 2015, the FMG model has been integrated by different types of primary care providers, including family physicians, nurse practitioners, nurses, social workers and pharmacists, among others. We collected data between January 2022 and April 2023. Respondents were asked to complete the anonymous e-survey on a voluntary basis.

The survey is based on a self-reported on-line reflective tool of 29 items on the practice of advanced access (*Outil Réflexif sur l’Accès Adapté*; ORAA) that was developed to enable the creation of a portrait on key pillars of the advanced access model.^14,15^ A companion tool was developed specifically for administrative officers with 17 items surveying their perspectives on advanced access strategies implemented or not (dichotomous answers in all cases). Surveys were available on-line in both French and English and took approximately 12 minutes to complete. The link was sent by e-mail to a contact person in each clinic who volunteered to participate in the project. Two reminders were sent by e-mail to the contact person along with the response rate of the different types of professionals working in the clinic. Each participant was assigned a unique identifier to ensure confidentiality through a double validation process. Upon completing the questionnaire, respondents received a personalized report with suggestions to improve their advanced access practice. Afterwards, respondents were invited to complete a short questionnaire to obtain continuing education credits from the FMOQ equivalent to one hour.

Descriptive statistics were generated for all items in the questionnaire that related to the degree of implementation of the advanced access model. Ninety-five percent confidence intervals (95% CI) were calculated where needed.

## Results

In total, 127 clinics across 14 regions of the province of Quebec responded. Of those, 999 family physicians, 107 nurse practitioners, and 411 administrative officers responded to surveys. From the 107 clinics that disclosed information on staff numbers for physicians and nurse practitioners, the response rate was estimated to be 62%. Similarly, for the 104 clinics that reported on the number of administrative officers, the response rate was 60%.

Table 1 shows the implementation level of various key principles recommended in advanced access, including strategies in place, assessments of supply and offer planning, measurements of access outcomes, and the use of strategies to respond to imbalances when needed.

**Table 1.** Implementation status of key principles of advanced access - n (%).

	Family physicians	Nurse practitioners
	n = 999^a^	n = 107^a^
Recommended strategies to enhance timely access		
Opening of schedule over 2 to 4 weeks	809 (82)	86 (83)
Reserve a minimum of 20% of appointment slots for semi-urgent and urgent conditions (within 48 hours) at all times	469 (48)	40 (39)
Access to the agenda for scheduling limited to a restricted number of people	590 (60)	46 (44)
Use of strategies to optimize appointment slot utilization	917 (93)	80 (78)
Scheduling over the phone consultations	912 (92)	97 (93)
Planning of supply, offer and recurring variations		
Use of a formula to estimate the supply (number of consultations requested) for the coming year	168 (17)	17 (16)
Use of a formula to estimate the offer (number of consultations offered) for the coming year	133 (13)	14 (13)
Consider variations in patient demand when planning service provision (e.g. flu season, summer period)	351 (35)	47 (44)
Planning of absences of more than 2 weeks	552 (80)	81 (95)
Strategies to react to offer-demand imbalances		
Use of a structured feedback mechanism in collaboration with officers responsible for making appointments	225 (23)	15 (14)
Measurement of the third next available appointment to notify of offer/demand imbalances	102 (10)	12 (12)
Use of strategies during peaks of demand	465 (47)	34 (32)

^a^Percentages were calculated based on the total number of responses received for each individual item.

### Recommended strategies to enhance timely access

One of the key strategies of the advanced access model, opening the schedule for appointments over a period of 2 to 4 weeks, was largely adopted by a total of 82% of respondents. However, another key strategy, planning to reserve at least 20% of consultation time for urgent or semi-urgent conditions, was implemented by fewer than 50% of respondents. Similarly, only 33% of the administrative officers surveyed reported using a referral algorithm to book appointments with the appropriate provider in a timely manner. Offering other modalities of consultation has also been highly integrated into regular practice, especially over-the-phone consultations (93%). Of note, limiting the number of people involved in appointment scheduling and strategies to optimize appointment scheduling or the consultation itself (e.g. eliminating annual routine exams for young patients, increasing intervals for patients with stable conditions, and renewing prescriptions for longer periods) were more frequently reported as strategies adopted by physicians than by nurse practitioners.

### Planning for supply, offer, and recurring variations

Planning for the supply and offer for the upcoming year based on the number and characteristics of the patient base was poorly implemented among both types of respondents (17% for supply and 13% for offer), with recurring variations being slightly better accounted for (36%). In contrast, planning for long absences (> 2 weeks) appeared to be well integrated, with better adherence by nurse practitioners (95%) than family physicians (80%).

### Strategies to react to offer-demand imbalances

Strategies to react to unforeseen offer-demand imbalances were insufficiently put in place by both types of respondents (45%), with low adherence to communication strategies about imbalances (22%) and strategies to identify imbalances, such as the third next available appointment (10%). Some of the main strategies normally used to respond to peaks in demand and avoid or minimize backlogs and wait lists (e.g. reducing length of appointments, increasing the number of appointment slots during the day, evenings or weekends, and add offering telephone or virtual appointments) appeared to have gained more traction among family physicians (47%) than nurse practitioners (32%).

## Discussion

For several years, attempts have been made to implement the advanced access model at a large scale across the province of Quebec with limited effects on patients’ perceptions of timely access to primary care.^4^ This study shows that some strategies rooted in the principles of the model have been implemented and integrated into the practices of family physicians and nurse practitioners, especially specific practices to improve agenda management. This implementation represents a first step. However, introducing a new model of practice as complex as advanced access requires a more global approach, both from professionals and organizations.

Several studies and systematic reviews on the effects of advanced access have shown variations in the implementation of the model across clinics and even among clinicians within the same organization.^6,9,16^ Most of these studies showed very uneven implementation of the different principles, with emphasis on same-day appointments diluting and confusing efforts to implement the model as a whole.^12,17-19^ The very few cross-sectional studies that have reported on strategies implemented showed fairly similar results to this study. Balancing supply with demand, identifying demand patterns, providing additional appointments to clear backlogs, and varying consultation modalities (mostly by telephone) are among the most implemented strategies across various settings.^20-23^ Advanced access strategies that are not widely used include involving patients in planning, telephone triage, planning replacements for absences, and the use of e-mail to correspond with patients.^20,22^ Measures to optimize consultations, to promote self-care (e.g. leaflets or web sites), and to improve interprofessional collaboration and shared care show conflicting results with respect to their implementation success.^20,22^

Advanced access strategies successfully implemented in our study included strategies based on individual practice change rather than changes typically implemented at the clinic level. Strategies requiring coordination within the team, major changes in care planning, or a rigorous communication plan showed lower degrees of adoption, likely due to their inherent complexity. Indeed, most of the strategies related to planning of offer and supply and strategies to rapidly identify and act upon offer-demand imbalances have still not been implemented by most respondents. Strong engagement of leadership in change initiatives, such as providing support to professionals for change management (e.g. a quality improvement committee, implementation plan, and change management training and guide) during implementation, has been identified as a key factor contributing to the success of implementing advanced access.^7,24,25^ Moreover, supporting clerical staff members by organizing ongoing weekly meetings focusing on problem solving was reported to be a crucial factor to success.^17^ Furthermore, that study showed that agreed-upon booking rules were not sustained when meetings were discontinued, suggesting a need to support adoption of the model at the organizational level throughout implementation.

The nature of the change required is not the only factor that contributes to implementation success; diffusion strategies also play a key role. Over the past few years, Quebec governing bodies have mainly opted for training and information initiatives to encourage implementation of the advanced access model. Simple change strategies such as educational meetings, audits and feedback, and printed educational materials have demonstrated some effects on behaviour change, but their success remains modest at best.^26^ Research shows that when complex interventions involving multiple stakeholders or organizations need to be implemented, simple “one-size-fits-all” strategies are not enough.^19,27^ As the efficacy of an intervention depends on the quality of the adaptations made to support the change and respond to the barriers faced, more intensive and continuous change interventions may lead to higher implementation levels.^19^ For example, practice facilitation relying on reflective practices can be used to tailor change strategies to local settings and contribute to higher buy-in from stakeholders.^7,24,25,28-31^

The success of implementation of an innovation requires continuous evaluation, either formally or in the form of reflexive feedback,^15^ which informs on what works and what does not. Structural barriers, such as a lack of data available in real time through electronic medical records, currently prevent such reflexive processes from taking place. This study revealed poor planning of services. A key principle of advanced access is based on achieving a supply-demand balance, and it is imperative to be able to evaluate this balance. Yet, professionals do not have access to data portraying their own portrait on access, such as their third next available appointment. Professionals need data to support supply planning based on the characteristics of their patients’ needs.

## Conclusions

This article demonstrates that few advanced access strategies have been successfully implemented. More initiatives are needed to achieve a sufficiently high rate of implementation of advanced access strategies to have an impact on the healthcare system. However, some recent quality improvement initiatives have shown very encouraging results at the clinic level.
